# Development of a Rapid Gold Nanoparticle-Based Lateral Flow Immunoassay for the Detection of Dengue Virus

**DOI:** 10.3390/bios12070495

**Published:** 2022-07-07

**Authors:** Cynthia Martinez-Liu, Carlos Machain-Williams, Natalia Martinez-Acuña, Sonia Lozano-Sepulveda, Kame Galan-Huerta, Daniel Arellanos-Soto, Mayra Meléndez-Villanueva, Diana Ávalos-Nolazco, Katya Pérez-Ibarra, Sergio Galindo-Rodríguez, Aurora de Jesús Garza-Juarez, Ana María Rivas-Estilla

**Affiliations:** 1 Department of Biochemistry and Molecular Medicine, Hospital Universitario “Dr. Jose E. Gonzalez”, Autonomous University of Nuevo Leon, Monterrey 64460, Nuevo León, Mexico; cmliu@outlook.es (C.M.-L.); nmartinez.me0120@uanl.edu.mx (N.M.-A.); slozano.me5017@uanl.edu.mx (S.L.-S.); kame.galanhr@uanl.edu.mx (K.G.-H.); daniel.arellanosst@uanl.edu.mx (D.A.-S.); mayra.melendezvl@uanl.edu.mx (M.M.-V.); diana.avalos31@hotmail.com (D.Á.-N.); katya.peerez@gmail.com (K.P.-I.); aurora.garzajr@uanl.edu.mx (A.d.J.G.-J.); 2 Laboratory of Arbovirology, Centro de Investigaciones Regionales “Dr. Hideyo Noguchi”, Universidad Autónoma de Yucatan, Merida 97000, Yucatan, Mexico; carmachain@gmail.com; 3 Department of Chemistry, Facultad de Ciencias Biológicas, Universidad Autónoma de Nuevo León, Monterrey 66450, Nuevo León, Mexico; sagrod@yahoo.com.mx

**Keywords:** dengue virus, gold nanoparticle, lateral flow assay, biosensor, immunochromatographic strip, point-of-care test (POCT), rapid test

## Abstract

Flavivirus detection in humans and mosquito reservoirs has been an important issue since it can cause a variety of illnesses and could represent a health problem in geographical zones where the vector is endemic. In this work, we designed and characterized a biosensor based on gold nanoparticles (AuNPs) and antibody 4G2 for the detection of dengue virus (DENV) in vitro, obtaining different conjugates (with different antibody concentrations). The AuNP–4G2 conjugates at concentrations of 1, 3, and 6 µg/mL presented an increase in the average hydrodynamic diameter compared to the naked AuNPs. Also, as part of the characterization, differences in the UV-Vis absorbance spectrum and electrophoretic migration were observed between the conjugated AuNPs (with BSA or antibody) and naked AuNPs. Additionally, we used this biosensor (AuNP–4G2 conjugate with 3 µg/mL antibody) in the assembly of a competitive lateral flow assay (LFA) for the development of an alternative test to detect the flavivirus envelope protein in isolated DENV samples as a future tool for dengue detection (and other flaviviruses) in the mosquito vector (*Aedes* *aegypti*) for the identification of epidemic risk regions. Functionality tests were performed using Dengue virus 2 isolated solution (TCID_50_/mL = 4.58 × 10^3^) as a positive sample and PBS buffer as a negative control. The results showed that it is possible to detect Dengue virus in vitro with this gold nanoparticle-based lateral flow assay with an estimated detection limit of 5.12 × 10^2^ PFU. We suggest that this biosensor could be used as an additional detection tool by coupling it to different point-of-care tests (POCT) for the easy detection of other flaviviruses.

## 1. Introduction

Dengue is a mosquito-transmitted virus and the leading cause of arthropod-borne viral disease worldwide. The dengue virus (DENV) causes a febrile disease with severe muscle spasms and joint pain. Although most cases are asymptomatic, severe cases and death can occur [1].

In the Americas, dengue incidence has increased from 1.5 million cumulative cases in the 1980s to 16.2 million in the decade between 2010 and 2019. The year 2013 was an epidemic year with 2 million cases recorded; 37,692 were severe and 1280 deaths occurred. In 2019, more than 3.1 million cases were registered, with 28,000 severe cases and 1534 deaths [2].

*Aedes aegypti* is the mosquito vector responsible for dengue propagation and is widely distributed in the Americas [3]. Therefore, approximately 500 million people are at risk of dengue infection [2], powered by the recent propagation of Zika in 2013 [4] and Chikungunya in 2015 [5]. This mosquito is also responsible for the transmission of other viruses such as Yellow Fever virus (YFV) and West Nile virus (WNV) and is considered the most important vector of human pathogen transmission [6].

Without any efficacious and fully licensed vaccines or treatment available against these diseases, vector control remains the immediate alternative to reduce or prevent transmission. Current national protocols in Mexico for vector control and transmission reduction include larvae control, entomological surveillance, and neighborhood spraying. Entomological surveillance incorporates molecular methods [7], usually quantitative real-time-PCR (RT-qPCR), for the identification, quantification, and genotyping of DENV and other arboviruses in mosquito populations [8,9,10,11,12]. However, this process requires a large logistic workflow from the collection of mosquitos around households to sample processing, nucleic acid isolation, and virus detection in authorized centers, requiring specialized reagents and equipment, well-trained personnel, and time.

Several tools have been designed as point-of-care tests (POCT) to improve current strategies even when no appropriate equipment or laboratory installations exist. POCT are performed outside of a laboratory, use mobile devices, and results are available in minutes. POCT developed for the detection of mosquito-borne viruses include the identification of proteins and viral RNA using gold nanoparticle biosensors [13,14], loop-mediated isothermal amplification (LAMP) [15,16], and most recently a portable device based on real-time PCR [17].

Among the different types of POCT, lateral flow assays (LFAs) are the simplest as they are easy to use without the need for specialized equipment. LFAs are paper-based chromatographic tests in which detection reagents are preloaded and activated when the sample is added, obtaining results in approximately 15 min. LFAs use gold nanoparticles (AuNPs) as labeling molecules that are functionalized with biological recognition elements to form specific biosensors that capture the antigens of interest to carry out the detection.

There are different design formats of LFAs; the selected format depends on the reagents that are available and the size and concentration of the analyte. Most LFAs are designed in a sandwich format using two antibodies that bind to different sites on the antigen to be detected (a capture antibody and an antibody immobilized in the solid phase of LFAs). In the presence of the analyte, two signals will appear in the test and control zone. In the competitive format, target analytes bind to reporters (recognition-labeling molecules) and block these reporters, preventing their binding to the ligand immobilized in the solid phase of LFAs. In the presence of the analyte in the sample, a single signal is shown (in the control zone) indicating that the analyte has bound to the reporter and formed a complex that binds to the secondary antibody immobilized in the solid phase of the LFA. This type of format is used when the analyte is small, the amount of analyte in the sample is very low, and/or the analyte has a single epitope for detection, which makes it impossible to use two antibodies in LFAs [18].

Recently, authors have reported the development of LFAs for virus detection based on the use of monoclonal antibodies and different labeling molecules such as gold nanoparticles and coated beads. These LFAs have been used for the identification of DENV and other flaviviruses in patient sera and isolated virus samples in order to improve the monitoring of these viruses [19,20].

In this work, we design and characterize a biosensor based on AuNPs and a commercial antibody commonly used in clinical diagnostics [21,22,23] (4G2 antibody) that specifically recognizes the domain II of protein E of the flavivirus group (including dengue virus, West Nile virus, Japanese encephalitis, Yellow Fever virus, Zika virus, etc.). In addition, we use this biosensor in the assembly of a competitive LFA for the development of an alternative test to detect the flavivirus envelope protein in isolated DENV samples as a future tool for dengue virus detection (and other flaviviruses) in the mosquito vector in order to identify epidemic risk regions. Also, we discuss the challenges and achievements reached during the design and testing of the biosensor and the competitive LFA.

## 2. Materials and Methods

### 2.1. Reagents

Citrate-coated spherical Gold Nanoparticles were purchased from Nanopartz (cat. A11-60-CIT-DIH), sucrose from Fermont (cat. PQ07642), and Tris from Biorad (cat. 161-0719). Bovine serum albumin (BSA), sodium azide (cat. 26628-22-8), sodium phosphate dibasic heptahydrate (Na_2_HPO_4_·7H2O) (cat. 7782-85-6), sodium dihydrogen phosphate monohydrate (NaH_2_PO_4_·H_2_O) (cat.10049-21-5), sodium tetraborate decahydrate (Na_2_B_4_O_7_·10H_2_O) (cat. 1303-96-4), triton X-100 (cat. 9002-93-1), sodium phosphate dibasic (Na_2_HPO_4_) (cat. 10890), polyvinyl alcohol (PVA) (cat. 341584-500g), polyvinylpolypyrrolidone (PVP) (cat. 25249-54-1), casein (cat. C6554), and tween 20 (cat. 9005-64-5) were purchased from Sigma-Aldrich.

Antibody against the flavivirus group, antigen 4G2, was purchased from Novus Biologicals (cat. NBP2-52709-0.2 mg) and goat anti-mouse IgG antibody was purchased from Abcam (cat. Ab6708). Absorbent pad (cat. 07.623.30), backing card (cat. 07.615.40), nitrocellulose membrane (NC) (cat. 07.626.10), sample pad (cat. 07.622.30), and conjugation pad (cat. 07.614.30) were obtained from Claremont Bio.

### 2.2. Dengue Virus Propagation

DENV was isolated from viremic patient serum. The viremic patient was sampled under routine care at Linares General Hospital; located in Linares County in Nuevo Leon, Mexico. The patient sought care in August 2012. The virus obtained from the sample was isolated and propagated in C6/36 cells, sequenced by the Sanger platform, and identified with the following access number in Genbank: KM279427.1. Viruses were propagated on C6/36 cells in Leibovitz L-15 media, supplemented with fetal bovine serum (5–10%), incubated at room temperature (25–28 °C) without a CO_2_ atmosphere in closed culture bottles, and purified by a traditional gradient-free ultracentrifugation protocol in a sucrose solution followed by selective virion precipitation as previously reported [24]. Viral isolates were titrated using the protocol for the formation of lytic foci or plaques in Huh-7 cells, stained with Naphtol blue-black dye and Median Tissue Culture Infectious Dose (TCID_50_), and the plaque-forming unit per mL (PFU/mL) was calculated by viral titration [25]; Equations and calculations can be viewed in Supplementary Information S1. Aliquots of viral stock were stored at −80 °C until use.

### 2.3. Preparation of Biosensor (Antibody-Conjugated AuNPs)

For the formation of the biosensor, the AuNPs were conjugated with the antibody 4G2 (cat. NBP2-52709-0.2 mg), a commercial antibody that specifically recognizes domain II of protein E of the flavivirus group (including dengue virus, West Nile virus, Japanese encephalitis, Yellow Fever virus, Zika virus, etc.). For conjugation, the pH of the AuNPs was adjusted to 6 by adding 0.1 M borate solution (pH 6), then, different final concentrations of antibody 4G2 (0, 3, and 6 µg/mL) were added into the AuNP colloidal solution (1 mL) with OD 1 (concentration of 0.05 mg/mL of AuNPs, according to the manufacturer). The mixture was incubated at 4°C for 40 min using a rotator. Following incubation, a conjugation solution (sucrose 2.1 M, BSA 0.1%, sodium azide 1%) was added and incubated at 4 °C for 10 min in the rotator. Then, 20 µL of 10% BSA was added and the mix was incubated for 10 min at 4 °C with shaking. The mixture of antibody-conjugated AuNPs was centrifuged at 2500× *g* for 15 min. The supernatant was discarded and the pellet was resuspended in 250 µL of storage solution (0.1 M sucrose, 0.1% BSA, and 0.01% sodium azide), concentrating the amount of AuNPs 4 times. The AuNP solution was stored at 4 °C until use.

### 2.4. Optimization of the Conjugation of Nanoparticles and GAT Value Calculation

To optimize the conjugation of antibodies on the surface of the AuNPs, the pH of the resuspended solution was changed before the addition of the antibody. To adjust the pH of the nanoparticle solution, a 0.1 M borate solution was used at a different pH (5.0–10.0). The stability of the conjugates at different pH values was measured by the gold aggregation test (GAT), modifying the absorbances used in the equation reported by Chamorro et al. [26] and adjusting the presented absorbance values for the 60 nm AuNPs. An amount of 100 µL of nanoparticles was placed in a 96-well plate, then 20 µL of 0.1 M borate buffer of each pH was added. An amount of 10 µL of the antibody (to a final concentration of 10 µg/mL) was added to the nanoparticles and incubated for 30 min under shaking at 4 °C. Each sample was prepared in triplicate and the absorbance value was measured at 534 and 600 nm in a spectrophotometer. Subsequently, a 20% NaCl solution was added to each well, incubated for 10 min at room temperature, and the absorbance at 534 and 600 nm was measured. The values were used to calculate the GAT value using the following equation, identifying the least stable conjugate as the one with the highest GAT value:

UAbs_Change_ = [UAbs534 − UAbs600]_Before NaCl addition_ − [UAbs534 − UAbs600]_After NaCl addition_ (1)

### 2.5. Characterization of AuNPs and the Biosensor AuNP–4G2

For the characterization of AuNPs and conjugates, an electrophoretic mobility test, colloidal gold aggregation test (GAT), and ultraviolet–visible absorption spectroscopy (UV–Vis) were performed and physicochemical parameters were determined (hydrodynamic diameter, ζ-potential, and polydispersity index) by dynamic light scattering (DLS) [27].

For the electrophoretic mobility test, 100 µL of the nanoparticles or the conjugates were centrifuged at 2500× *g* for 15 min at 4 °C. The pellet was recovered and resuspended in 20 µL of 0.1 M borate buffer pH 8 and mixed with the loading buffer (Tris HCl pH 8, 40% glycerol, and bromophenol blue) and loaded in 1% agarose gel (0.5X TBE). The gel was run in 0.5% TBE buffer for 1 h at 150 V and 200 mA.

To identify the resistance to aggregation of the nanoparticles and conjugates due to antibody/albumin adsorption, the gold aggregation test (GAT) was performed as mentioned above.

Ultraviolet−visible spectra of the AuNPs and AuNP conjugates were measured with a spectrometer Microplate reader Cytation 3 (Biotek Instruments). In a 96-well plate, 100 µL of the AuNPs or conjugates (24 h after conjugation) were placed into and read by the equipment. The samples were read in triplicate in the spectrophotometer in a range of 450−700 nm wavelength every 2 nm and the averages were plotted.

Hydrodynamic size, ζ-Potential, and polydispersity index of AuNPs and conjugates were determined using a DLS Zetasizer Nanoseries (Marvern instruments, Malvern, UK) with a 4 mW laser (633 nm) at 25 °C. To prepare the samples, 100 µL of the AuNPs and conjugates (24 h after being prepared in storage solution) were placed in a cell (quartz to measure size or disposable capillary cell for ζ-potential), 900 µL of deionized water was added, and the samples were read by the equipment. Each measurement consisted of 20 runs and samples were analyzed in triplicate.

### 2.6. Preparation and Assembly of the Lateral Flow Assays

**Figure 1:**
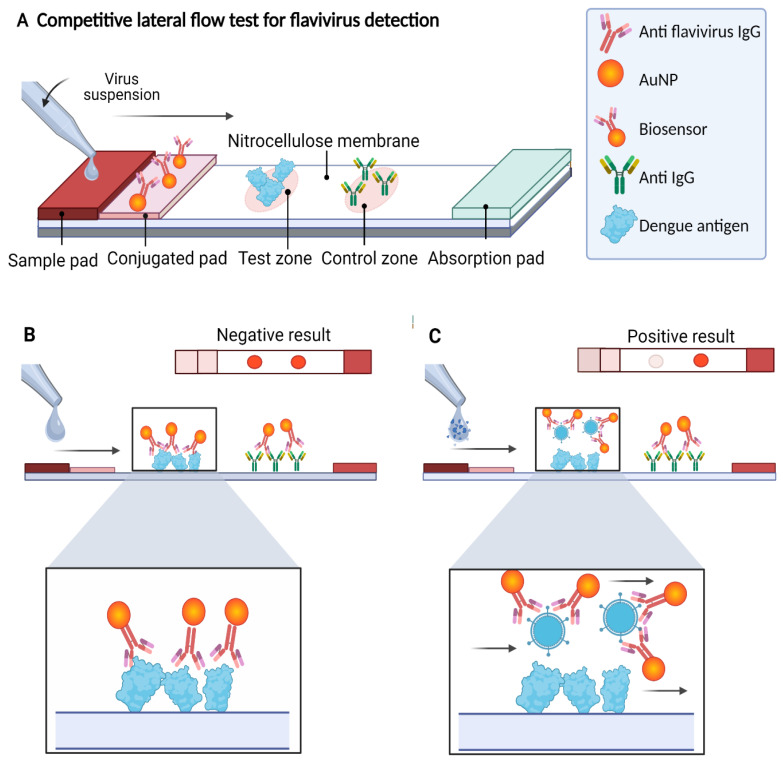
Scheme of the competitive lateral flow assay designed in this study for the detection of flaviviruses. (**A**) On the strip, the sample flows by capillarity to the different components, reacting with the biosensor on the conjugate pad producing a signal on the detection membrane. (**B**) If the sample does not contain flaviviruses, the biosensor flows to the detection membrane, binding to the analyte present in the test zone (DENV) and the secondary antibody against IgG present in the control zone, generating two signals (negative test). (**C**) If the sample contains flaviviruses, the biosensor–analyte complex is formed, which flows to the detection membrane, generating signals only in the control zone (positive test). In both cases, the signal from the control zone must be observed to confirm correct operation of the LFA.

In this work, we developed an LFA in a competitive format in which the presence of the analyte (the virus antigen) from the sample “competes” to bind to the biosensor with the analyte present in the test zone of the detection window. The signal in the test zone indicates the absence of the target analyte in the sample, whereas the signal in the control zone indicates that the biosensor flowed correctly through the LFA. Therefore, the design allows two signals to be observed when the test is “negative” for the presence of viral antigen, whereas one signal (only in the control zone) is observed when the test is “positive”, indicating the presence of the viral antigen in the sample. Due to the AuNPs used in the biosensor preparation, the signals are visible to the naked eye as reddish spots (Figure 1).

The LFA consists of four components pretreated and mounted together on an adhesive backing card: a sample pad, a conjugation pad, a nitrocellulose membrane (detection membrane), and an absorbent pad. The performance diagram and the components of the LFA are shown in Figure 1A. Each component was prepared separately as follows and then mounted on an adhesive backing card before use.

The sample pad was previously treated with a solution of 0.1 M sodium tetraborate decahydrate, 0.05% triton, and 0.5% BSA adjusted to pH 8 for 5 min in rotary shaking, allowed to dry for 2 h at 37 °C, and stored at 4 °C until use.

The fiberglass conjugation pad was treated with a solution of 0.05 M dibasic sodium phosphate, 0.05% triton, 0.5% BSA, and 0.5% polyvinyl alcohol pH 7.4 for 5 min under stirring and allowed to dry at 37 °C for 2 h. Subsequently, the AuNP–4G2 (3 µg/mL) conjugates were applied to the conjugation pad until saturated and allowed to dry for 30 min at 37 °C.

For the detection membrane, a nitrocellulose membrane (Nitrocellulose FF 170) was used. Solutions were prepared as follows: DENV antigen undiluted and goat anti-mouse IgG secondary antibody were diluted separately with 1:1 in a 1% methanol–PBS solution. DENV antigen solution was immobilized on the membrane as a dot in the test zone, whereas the secondary antibody solution was placed as a dot in the control zone. Both were allowed to dry at room temperature for 10 min and subsequently, the membrane was blocked with a TBS solution with 0.015% casein, 0.3% polyvinylpyrrolidone, and 0.001% tween 20 pH 8 for 10 min under stirring at room temperature and allowed to dry at room temperature.

The cellulose absorption pad had no treatment. Each of the dry components was cut into 10 cm rectangles, assembled on the adhesive backing card, and placed in the Matrix 1201 membrane cutter from Kinematic Automation to form the strips. The LFAs were stored at 20–22 °C in sealed packages containing silica gel.

### 2.7. Performance Test of the LFA Using AuNP–4G2 Biosensor

To determine the in vitro functionality of the LFA, PBS buffer 10 mM NaCl was used as a negative control and the DENV-isolated solution at a TCID_50_/mL = 4.58 × 10^3^ (3.2 × 10^3^ PFU/mL) as a positive sample. Before adding the sample, the LFAs were allowed to dry for 10 min at room temperature and then 160 µL (5.12 × 10^2^ PFU) of the DENV-positive sample was placed on the sample pad and allowed to migrate through the strip by capillarity. After 10 min, the results were observed in the detection window. According to the design of the LFA, two reddish spots (one in the test zone and the other in the control zone) were interpreted as negative (see Figure 1B) and the LFAs with only one reddish dot (in the control zone) were interpreted as positive (see Figure 1C).

### 2.8. Statistical Analysis

We compared the AuNP control and conjugates using one-way analysis of variance (ANOVA) and Tukey’s multiple-comparison post-test. Differences between groups were significant at a *p* value of <0.05. Statistical analyses and graphics were made with GraphPad Prism 6.0 (GraphPad Software, Inc., San Diego, CA, USA).

## 3. Results

In this work, we designed a competitive POC detection system formed by a biosensor based on AuNPs coupled to specific antibodies against protein E present in the Flaviviridae family that detects the antigen forming a AuNP–antibody–antigen complex and the assembly of different components that allow the flow and visible signal detection in the LFA in 10 min. With no antigen in the analyzed sample, the biosensor is free to bind to the antigen present on the detection membrane, specifically in the test zone. In addition, as a test control, a secondary antibody (goat anti-mouse IgG antibody) that binds to the primary antibody (antibody against flavivirus group antigen 4G2) used in the biosensor was added to the control zone of the detection membrane to ensure that the AuNPs are coated with the detection antibody and have also migrated correctly through the LFA.

This work is divided into three main parts: first the preparation and characterization of a biosensor that recognizes the antigen (DENV), second, the design and assembly of the LFA, and third, the assessment of the functionality and the optimization of the LFA.

### 3.1. Preparation and Characterization of the Biosensor

To prepare our biosensor, citrate-coated AuNPs of 60 nm were conjugated by adsorption with a 4G2 antibody. First, we determined the optimal conditions for the conjugation process. For this purpose, the pH of the nanoparticle solution was adjusted from 5 to 10. After that, AuNPs were conjugated with a final antibody concentration of 10 µg/mL (maximum concentration of antibody). The optimal pH for conjugation was determined by measuring the aggregation capability of the conjugated AuNPs. Each AuNP–antibody conjugate was incubated with a 20% NaCl solution where the saline environment generates negative charges in the medium and increases the interactions between AuNPs with negative charges available on their surface (not completely covered by the antibody), resulting in the aggregation of the AuNPs and the formation of agglomerates. If the conjugation conditions did not allow the antibodies to bind to the surface of the AuNPs and cover it completely, aggregates will be generated in the presence of sodium chloride. The formation of these aggregates was measured in each condition tested during the conjugation process by the gold aggregation test (GAT). In this test, they measured the absorbance values at 534 and 600 nm before and after the addition of NaCl to the AuNP solution (at different pH values) after conjugation with the antibody. The values were used to calculate the number of GATs from the formula established by Chamorro et al. [26]; the lower the value of the GAT number, the better the coating of the surfaces of the nanoparticles with the antibodies, indicating the best condition for conjugation.

**Figure 2:**
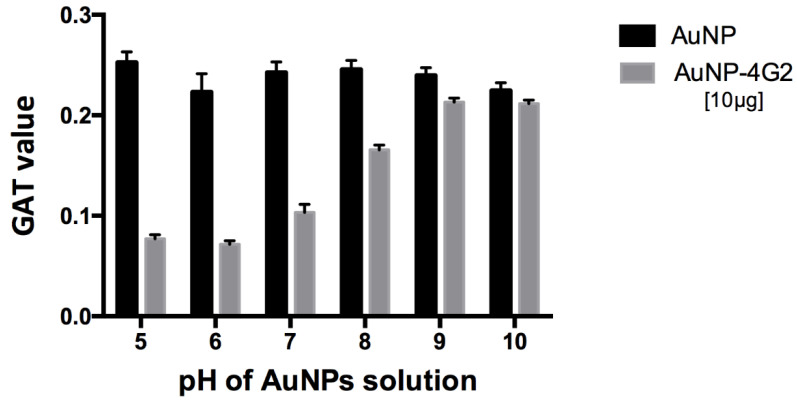
Optimization of pH conditions for the conjugation of antibodies on the surface of AuNPs. For the 60 nm citrate-coated AuNPs used, the optimal pH of the conjugation was determined using the gold aggregation test (GAT) to establish at which pH the minimum value was obtained (less aggregation, greater stability), using a maximum concentration of 4G2 antibody (10 µg/mL). The results indicated a minimum aggregation value of 0.0715 at pH 6 compared to the other values reported using a pH from 5 to 10 for AuNP–4G2, whereas the naked AuNPs presented aggregation in the presence of salts at any pH tested.

For the 60 nm citrate-coated AuNPs used, pH 6 was determined as the best for conjugation with the 4G2 antibody, showing a minimum aggregation value of 0.0715 compared to the other values obtained at pH 5–10, which were between 0.077 and 0.213 (Figure 2).

**Figure 3:**
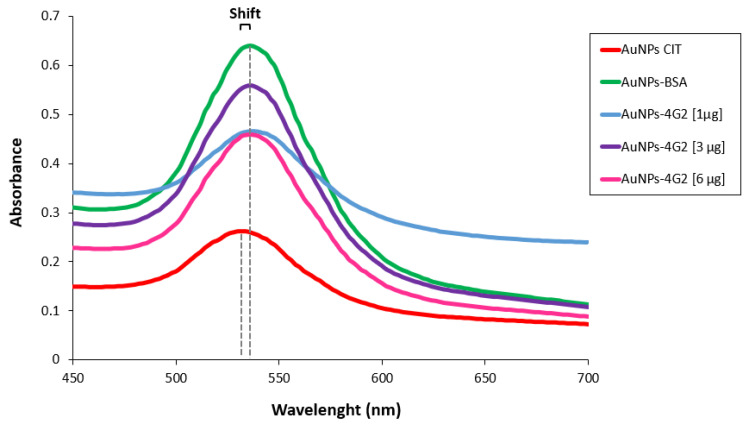
Characterization of AuNPs and AuNP–4G2 conjugates. UV-Vis spectra of AuNPs with citrate without treatments (AuNP CIT), AuNP–BSA conjugates (control), AuNPs conjugated with different concentrations of anti-flavivirus antibody of 1, 3, and 6 µg/mL (AuNP–4G2 conjugates). A shift in the maximum peak absorbance of the spectrum was observed in the AuNP–4G2 conjugates at different antibody concentrations and in the AuNP–BSA conjugates compared to the naked AuNPs (the maximum absorbance peaks are indicated by the dotted lines).

Once the best conditions for conjugation have been determined, the concentration of antibody used for conjugation was optimized using 1, 3, and 6 µg/mL to coat 1 mL of citrate-coated gold nanoparticles (OD 1). As a conjugation control, BSA was used to coat the AuNPs (AuNP–BSA). The conjugates obtained (AuNP–4G2), the control (AuNP–BSA), and the unconjugated AuNPs (AuNP CIT) were characterized to identify the minimum concentration of antibody for coating the nanoparticle surface that generates the stability of the AuNPs in a colloidal state. Various techniques were used to characterize the conjugates and evaluate their properties. First, the absorbance spectrum band of the surface plasmon of the AuNPs and conjugates was determined by a spectral scan performed in the UV-visible range of 450 to 700 nm by spectrometry (see Figure 3). The UV-Vis spectrum of the AuNP CITs (as purchased) shows a spectral band with a maximum absorbance peak at 534 nm, whereas the AuNP–4G2 (the three conjugates) and AuNP–BSA presented a maximum absorbance peak at 536 nm. This shift in the value of the maximum peak indicated the configuration of the AuNP surface, going from a bare state to a coated state.

**Figure 4:**
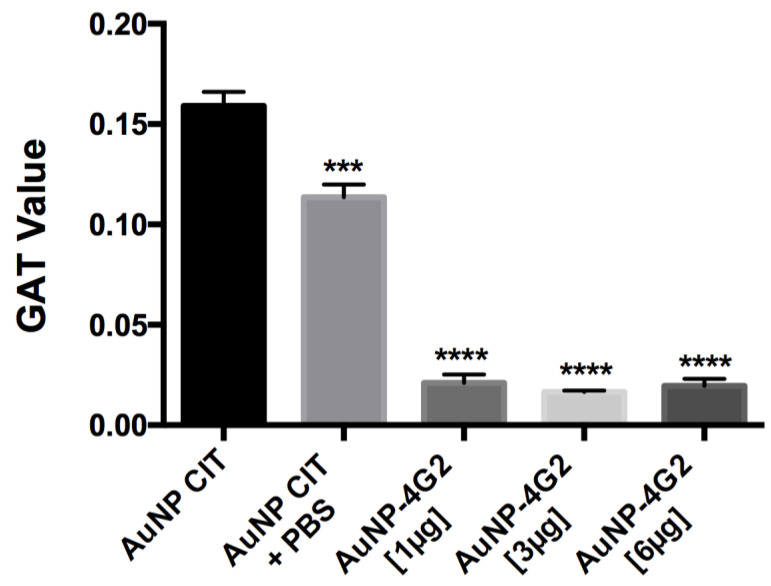
Determination of the aggregation value of the AuNPs and conjugates. The aggregation value was calculated using the gold aggregation test (GAT). All the conjugates presented values lower than those reported in the naked AuNP indicating the presence of the surface coverage of the AuNP by the antibodies, protecting the AuNP from the formation of conglomerates and preserving stability in the colloidal state. (*** *p* < 0.001, **** *p* < 0.0001, *n* = 3).

To corroborate the state of aggregation of the AuNP–4G2 conjugates, the GAT assay was performed. The results showed that the naked AuNPs (AuNP CIT) in the presence of PBS and borate buffer pH 6 reported a high aggregation number (<0.159), whereas the AuNP–4G2 conjugates presented values lower than 0.02, indicating their stability even in the presence of a high concentration of salts (see Figure 4). Among the AuNP–4G2 conjugates, it was observed that AuNP–4G2 conjugated with 3 µg/mL presented a lower aggregation value compared to AuNPs without coating or conjugated with 1 µg/mL of the antibody. Additionally, no major difference was observed between the aggregation value of the AuNP–4G2 conjugates with 3 and 6 µg/mL, so it was decided to work with a concentration of 3 µg/mL to conjugate the AuNPs and obtain the biosensor that was used in the LFA for the detection of flaviviruses.

For the physicochemical characterization, dynamic light scattering (DLS) was used to determine the hydrodynamic size, ζ-potential, and state of aggregation of the AuNPs and conjugates. The values of each measured parameter can be seen in Table S2 in the Supplementary Materials.

**Figure 5:**
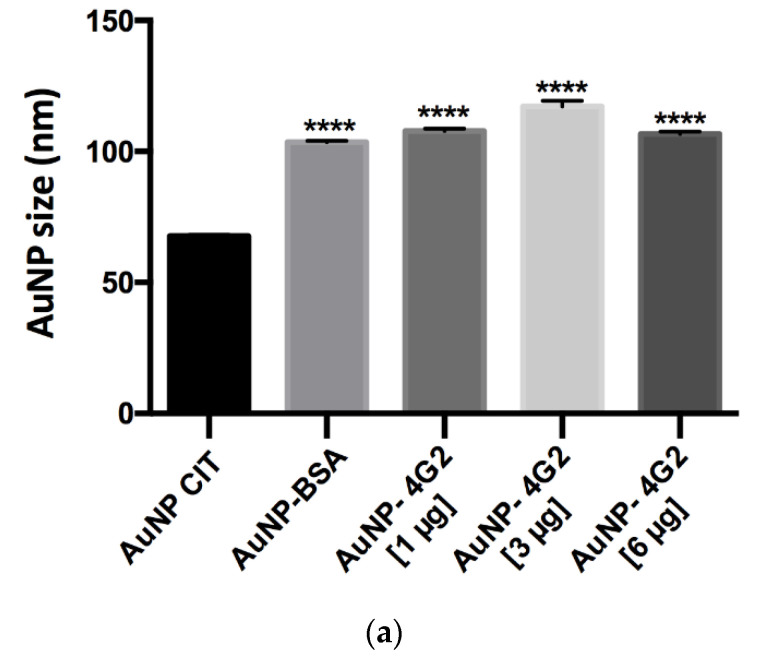

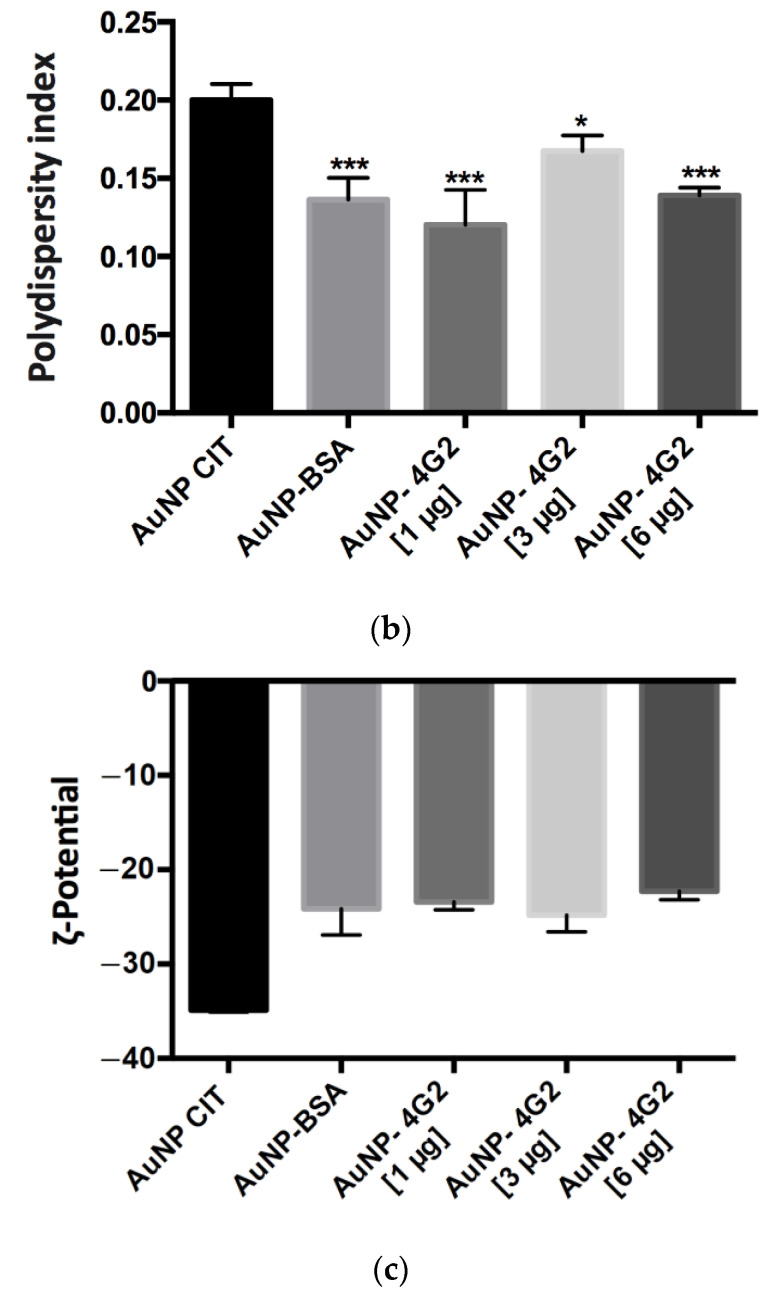
Physicochemical analysis of AuNPs and conjugates by DLS. (**a**) Hydrodynamic diameter (size), (**b**) polydispersity index, and (**c**) ζ-potential values of the naked AuNPs (AuNP CIT), control (AuNP–BSA), and conjugates (AuNP–4G2) (* *p* < 0.05, *** *p* < 0.001, **** *p* < 0.0001, *n* = 3).

The results obtained showed that the AuNP CIT presented an average hydrodynamic diameter of 67.69, whereas the control AuNP–BSA and the AuNP–4G2 at concentrations of 1, 3, and 6 µg/mL presented an increase in the average hydrodynamic diameter (size) of 103.5, 107.87, 117.20, and 103.63, respectively. The increase in the size of the conjugates and the control indicates the formation of the AuNP–antibody complex. However, the maximum increase in the AuNP size was observed in the AuNP–4G2 conjugate at an antibody concentration of 3 µg/mL, indicating that this concentration is the optimal one for the saturation of the nanoparticle surface (see Figure 5a).

On the other hand, the measured polydispersity value was less than 0.2, which indicated that the AuNP–4G2 and the control were monodisperse in a colloidal state and did not present aggregation, which is ideal for use in the detection of the analyte (Figure 5b).

When analyzing the ζ-potential values obtained, we observed that the AuNP CIT (naked) presented a value of −34.9 mV, a lower value than the 4G2–AuNP conjugates, indicating the successful deposition of antibodies on the surface of the nanoparticle and preserving stability (Figure 5c).

**Figure 6:**
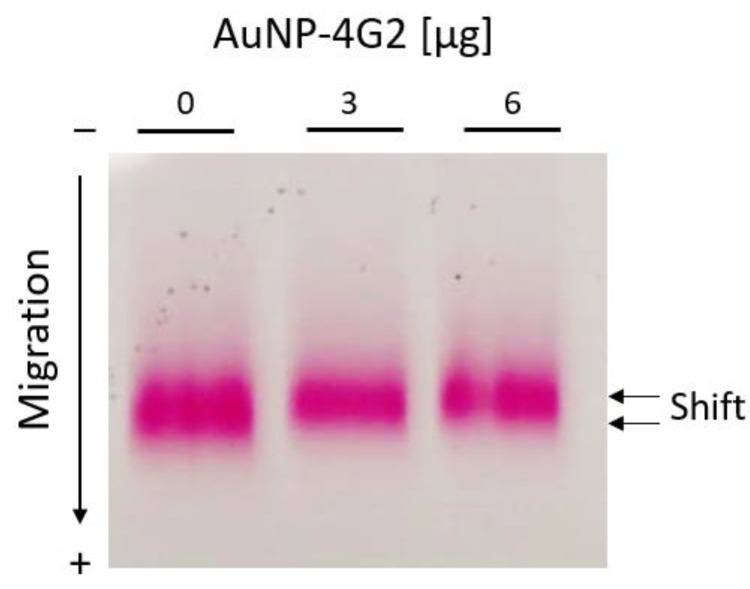
Electrophoretic mobility shift assay for AuNP–BSA and AuNP–4G2 conjugates. Agarose gel electrophoresis was used to identify the migration differences of each conjugate of AuNPs with increasing concentrations of anti-flavivirus antibodies (0, 3, and 6 µg/mL).

As complementary tests for the characterization of the AuNPs and AuNP–4G2 conjugates, the electrophoretic mobility test and the gold aggregation test were performed. According to the results observed in Figure 6, the AuNPs performed a delay in mobility when they were coated with 3 and 6 µg/mL of antibody compared to the controls (BSA, without antibody). This change in mobility suggests a larger size of the conjugated AuNP–4G2 due to slower migration.

### 3.2. Lateral Flow Assay Design

**Figure 7:**
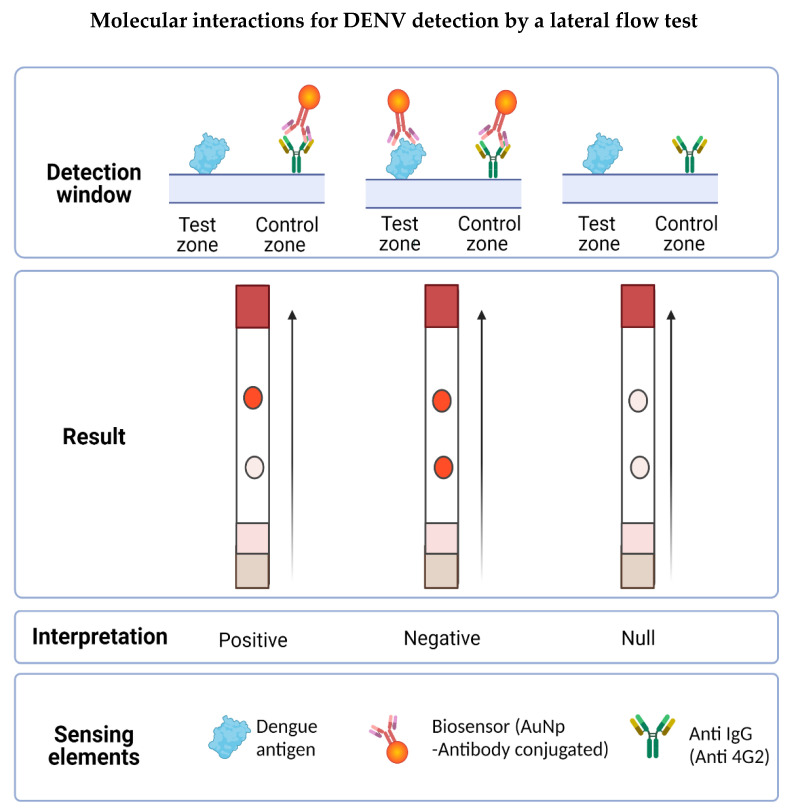
Molecular interactions involved in the possible results obtained in the LFA. The detection window is made up of the test zone and the control zone. In the first, the DENV is immobilized whereas in the second, the secondary anti-IgG antibody is immobilized. When placing the sample, the flavivirus present interacts with the biosensor forming a complex, which prevents it from joining the test zone but is recognized by the control zone, obtaining a positive result (a signal). If the sample does not present the antigen to be detected, the biosensor flows to the detection window and forms a complex with the virus present in the test zone, also binding to the antibody in the control zone, obtaining a negative result (two signals). Results will be null when the test zone has no signal, indicating that the biosensor did not flow properly on the LFA.

The LFA designed in this work was based on the competition of the antigen present in the tested sample with the antigen anchored to the nitrocellulose membrane by binding to the biosensor for the detection of flaviviruses. In the presence of flaviviruses, the biosensor forms a complex with the antigen present in the sample and is unable to bind to the previously immobilized antigen on the strip, but it can bind to the secondary antibody (into the control zone), observed by a single red dot. In the absence of flaviviruses in the sample, the biosensor binds to the test zone and the control zone, resulting in a negative test showing two red dots (see Figure 7).

**Figure 8:**
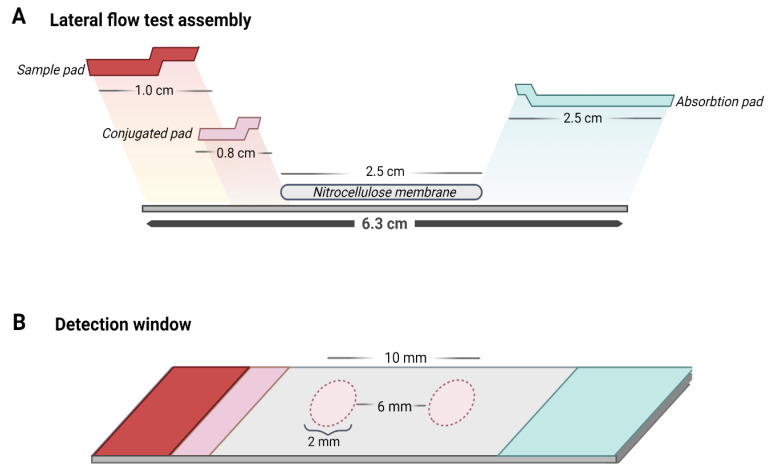
Design of the lateral flow assay for the detection of flaviviruses. (**A**) From left to right, the strip is composed of a sample pad, conjugate pad, detection membrane (with a test zone and a control zone), and absorbent pad. Each of the overlapping components as shown in the image have specific measurements that allow the flow in the strip for the formation of biosensor–virus complexes for detection. (**B**) The detection membrane is formed by the test zone and the control zone, where each one has a defined size that makes it possible to observe the results with the naked eye and the separation between them, with a constant measure, ensures the results of both zones do not overlap.

To optimize the LFA, different measurements of the materials that compose it were tested. In addition, the overlap distance between the materials was modified to obtain a better flow in the strip and therefore improve the formation of complexes for detection. Finally, the exact measurements of the LFA were determined as shown in Figure 8A,B. Additionally, it was established that the minimum volume of sample to obtain a result was 160 µL.

### 3.3. Performance and Reproducibility Tests of Lateral Flow Assay

**Figure 9:**
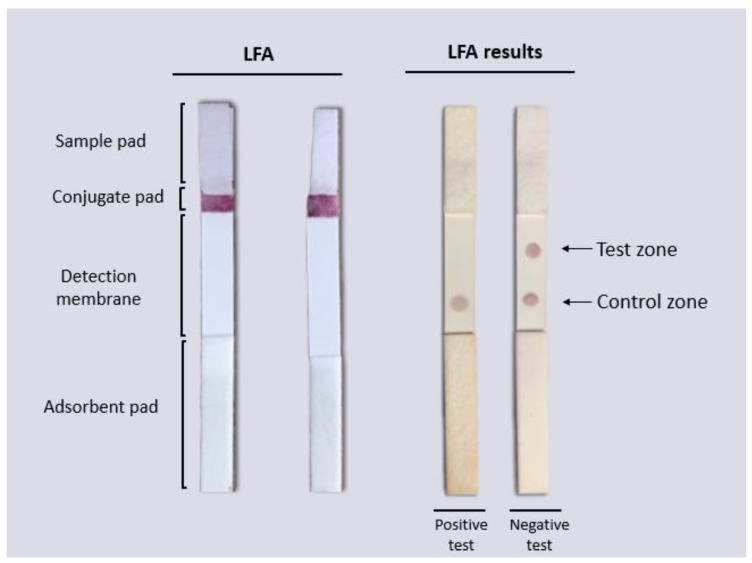
Functionality test of the lateral flow assay for the detection of flaviviruses. PBS with DENV (+) and PBS (−) were used for the LFA functionality test. The biosensor placed on the conjugate pad, upon contact with the sample, was able to recognize the virus, form a complex, and not bind to the analyte (DENV) bound to the membrane in the test zone, producing a visible red signal in the control zone by binding to the biosensor antibody 4G2 with the membrane-attached IgG secondary antibody.

To test the functionality of the LFAs, 160 µL of each sample (DENV isolated as a positive sample and PBS buffer as a negative sample) was used. Samples (positive or negative) were placed on the sample pad of the LFA and allowed to run at room temperature and after 10 min, the results were observed and photographic evidence was taken. The results can be observed in Figure 9. In the LFAs where positive samples were analyzed, a single signal was observed in the control area, indicating the union of the virus with the AuNP–4G2 conjugates (forming a complex), and preventing the AuNP–4G2 conjugate from binding to the immobilized virus in the test zone. Furthermore, a signal was observed in the control zone where the conjugates (forming a complex with the antigen or not) bound to the immobilized anti-mouse antibody. The LFAs where the negative samples were placed (which contained only PBS buffer) showed two signals in the detection window (in the test area and the control area), one indicating the binding of the AuNP–4G2 conjugate to the immobilized virus in the test zone and the other the recognition of the conjugate to the immobilized anti-mouse antibody in the control zone.

**Figure 10:**
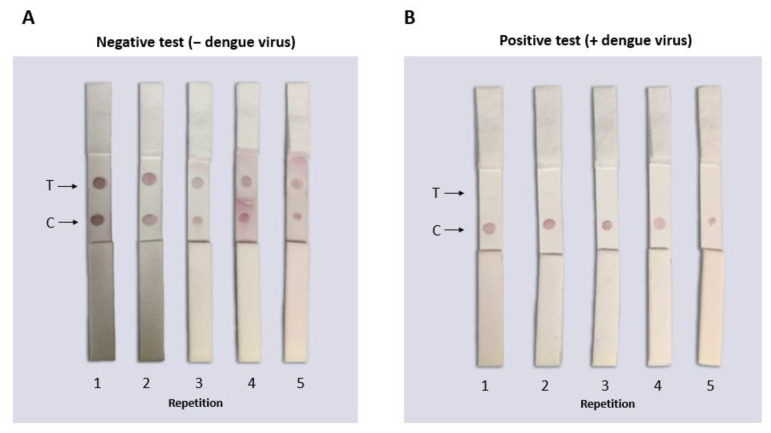
Functional reproducibility test of the lateral flow assay for the identification of flaviviruses. The tests were carried out with the same batch of tests assembled under the same conditions using PBS buffer for the negative sample or DENV for the positive sample (160 µL total volume). (**A**) In the LFAs where negative samples (only with PBS buffer) were placed, two red dots (signals) were observed indicating the absence of DENV. (**B**) In the LFAs where positive samples were placed (PBS and DENV), a single signal was observed in the control zone where the DENV in the sample “competed” with the virus immobilized in the LFA to bind to the biosensor. No false positives or negatives were observed in any of the tests performed.

Fifteen LFAs from the same batch were serially tested to observe detection reproducibility under the same conditions. Figure 10A shows the representative samples from the same batch testing negative samples that did not have DENV (only PBS buffer 10 mM NaCl). It was observed that the PBS buffer 10 mM NaCl was optimal to use as a run buffer for this test because none of its components generated false negatives or caused aggregation of the conjugates. Figure 10B shows the representative samples of the batch of LFAs tested with PBS and DENV where a single dot (in the control zone) corresponding to a positive result was observed. The consistency of the results showed that the tests from the same batch had the same characteristics and could detect the virus in vitro.

## 4. Discussion

Surveillance of flavivirus outbreaks has been of utmost importance due to the severity of the clinical presentation that can occur during acute infection in humans and other vertebrates. In addition, the epidemic potential of flaviviruses is increased by the geographic expansion of the vectors. The creation of ideal habitats for their reproduction constantly threatens public health and highlights the need for monitoring tools for the prevention and containment of outbreaks. In response to this need, in this work, we proposed a fast, portable, and low-cost tool for the identification of DENV and other flaviviruses using a POC test that uses a biosensor composed of gold nanoparticles and antibodies for detection.

This biosensor was made by functionalizing 60 nm of spherical gold nanoparticles with anti-flavivirus antibodies using passive adsorption conjugation. The size and shape of the AuNPs are crucial in determining the sensitivity of the detection. In terms of size, it has been reported that AuNPs with sizes between 20 and 40 nm are the most used for detection in LFAs [28,29]. However, a recent study in 2017 by Zhan et al. [30] compared AuNPs of 40, 60, and 100 nm, and showed that they were able to improve analytical sensitivity by up to 256 times when 60 and 100 nm AuNPs were used for the formation of the biosensor with antibodies. This is because the larger the size, the greater the surface area available for binding to antibodies, improving the detection of the analyte. Other authors have used and evaluated the functionality of the 40–60 nm AuNPs for the development of biosensors for use in LFAs [31,32].

Also, in terms of shape, spherical AuNPs have been widely used in the detection of analytes in LFA tests due to their unique optical and physicochemical properties, high biocompatibility, tunable monolayer, controlled dispersion, high surface area for functionalization with detection elements, low toxicity, high stability, and their characteristic red color that allows straightforward detection of complex formations without the need for extra equipment.

On the other hand, the antibody used for the formation of the biosensor (4G2 antibody) has been widely used in flavivirus detection assays and has recently been used as a control in dengue virus neutralization assays [33] and Zika immunodetection [34]. Its binding specificity to protein E of viruses of the *flaviviridae* family such as dengue (the 4 serotypes including serotype 2 used in this work) [35,36], Zika [37], West Nile virus [38], and tick-borne encephalitis virus (TBEV) [39] has been reported by multiple authors over time.

To ensure the functionalization of AuNPs, the conjugates (4G2–AuNP) were subjected to various physicochemical characterization tests. The results were compared with the naked nanoparticles (AuNP CIT), obtaining differences between the properties of the conjugates with respect to the unconjugated AuNPs to establish a pattern of conjugation control. First, the optimal conditions for the formation of the AuNP–antibody conjugates were determined using a set concentration of anti-flavivirus 4G2 antibody (10 µg/mL), varying the pH of the AuNP solution just before placing the antibody during the protocol of conjugation. In previous studies, it has been reported that the pH during conjugation determines the binding orientation of the antibodies in the AuNPs and can influence the optimal coating of their surface [40].

Subsequently, the stability of each conjugate formed at different pH values was tested using the GAT. The optimal pH for these conjugates was determined to be 6, where a lower aggregation of the AuNPs was observed in the presence of salts. The correct coating of the AuNPs ensures their monodisperse state that favors the uptake of the antigen by the biosensor and the formation of individual AuNP–antibody–antigen complexes, which can be used in an LFA or other detection systems.

Although using a high concentration of antibodies stabilizes the nanoparticles and increases the probability of the correct orientation of the antibodies on the surface, it has been reported that it can also cause the inaccessibility of the antibodies due to the effects of superposition and, therefore, could compromise its ability to detect the antigen [41].

Therefore, once the conjugation protocol was optimized, the optimal concentration of the antibody required for biosensor formation was determined. For this, three different concentrations of the antibody (1, 3, and 6 µg/mL) were tested for the formation of the conjugates. Each conjugate was characterized and its stability was analyzed by physicochemical parameters and its resistance to aggregation by the GAT test.

It was observed that the three AuNP–4G2 conjugates in the spectral scan UV-visible range presented a shift in the maximum absorbance peak compared to the naked AuNPs. This shift in the maximum absorbance peak of the conjugates has been previously reported as being indicative of the adsorption of the antibodies on the surface of the AuNP forming a monolayer [42].

Additionally, the hydrodynamic diameter of the conjugates was measured by DLS. An increase in the hydrodynamic diameter (relative size) proportional to the increase in the antibody used for conjugation was observed, indicating that at a higher concentration of the antibody, the formation of the monolayer in the AuNP increased. However, when 6 µg/mL of the antibody was added, a decrease in the hydrodynamic diameter was observed. These results may indicate the supersaturation of antibodies on the surface of the AuNP where at higher concentrations of the antibody there is competition or steric hindrance to binding to the surface.

Other parameters such as the ζ-potential and polydispersity index of the conjugates were compared between conjugates and with the naked AuNPs. It was observed that the increase in the ζ-potential measured in the AuNPs coincides with the addition of antibodies on the surface of the AuNP when forming the conjugates, suggesting that the negative charge that the AuNP surface possesses decreased as the AuNPs formed ionic bonds with the antibody.

The values obtained in the physicochemical parameters showed that the three conjugates presented similar characteristics, so in the last differentiation step, an aggregation test was performed. This test measured the stability of the conjugates in saline solution (20% NaCl) where a considerable difference was not observed between the stability of the conjugate with 3 and 6 µg/mL (GAT values less than 0.02), indicating that from 3 µg/mL, the surface of the AuNPs has an antibody monolayer that prevents aggregation. These tests have been performed by other authors in order to determine the minimum concentration of antibodies for the coating of AuNPs [43,44]. Therefore, the biosensor that was used to assemble the LFA was 3 µg/mL.

The LFA reported in this work was assembled with a biosensor using a competitive format to identify dengue (and other flaviviruses). This format is based on the fact that the antigen immobilized that is present in the detection zone “competes” with the antigen of the sample to bind to the biosensor. However, initially, we designed both formats, direct or sandwich-type and competitive type using the same biosensor, where under similar conditions we did not achieve the detection of the analyte using the sandwich-type. It has been reported that sandwich-type LFAs are typically used for larger analytes with multiple antigenic sites using different antibodies in the design [45].

The first LFA design was a sandwich-type using the same antibody both for the functionalization of the AuNPs and the elements immobilized on the detection membrane. In the functionality tests performed, we could not observe any signal in the test zone when we added DENV to the carrier solution (PBS) but a signal was observed in the control zone, indicating that the sample migrated correctly through the LFA. In order for the LFA to be functional and detect the analyte, we changed the treatments of each pad hoping that the detection would improve; however, after several optimization attempts, there was no positive result. There are multiple works where the sandwich format is used for viral detection; however, in the detection system reported in this work it did not work.

Then, with the same biosensor and the DENV as an immobilized analyte in the test zone, an LFA with a competitive format was designed. Subsequently, the LFA was tested in vitro to determine its functionality with DENV samples and the carrier solution (PBS). It was observed that in a batch of LFAs when the DENV positive sample was placed in the test, a signal was generated in the detection window, demonstrating that the virus was binding to the biosensor and preventing binding to the virus immobilized in the membrane. The LFAs where only the carrier solution was tested, showed two signals indicating that the biosensor bound to the antigen immobilized on the membrane, therefore indicating a true negative. The LFA designed with this AuNP-based biosensor allowed the detection of DENV in vitro; however, it is worth mentioning that this test can detect various flaviviruses due to the nature of the antibody (anti-flaviviruses 4G2) used for biosensor formation, so it could be included as an accessible option for flaviviruses monitoring.

In this work, the biosensor that we used in the LFA contained ~0.2 mg/mL of gold nanoparticles. It has been reported that the concentration of AuNPs can enhance the detection of the analyte in the LFA. However, a recent study published by Khlebtsov et al. in 2019 [46] indicates that although it is true that the signal obtained in the test zone decreases as the number of applied particles decreases, the amount of the signal observed in the test zone and the LFA limit of detection (LOD) are mainly determined by the light absorption of the particles used, which also depends on its size and shape.

The results found in this work offer this LFA as an alternative method for the detection of pathogens from the transmitter vector. In 2018, Basso et al. [47] generated a similar DENV detection system (serotypes 1–4) using a biosensor with antibody-coated nanoparticles, determining the presence of the virus through the physicochemical changes observed in the nanoparticle. Despite its advantages, the disadvantage of this method is the specialized equipment required for the analysis of each sample, making it not feasible as a field test compared to our portable test for which reading equipment is not necessary.

Other methods based on biosensors composed of AuNPs coated with other molecules, such as DNAzymes [48], have been used for the identification of arboviruses in the transmitting mosquito; however, these are usually complex to produce (and therefore expensive) and require an environment favorable for the development of the reaction, which makes it difficult to handle in an uncontrolled environment.

Regarding the detection limit, the authors usually report this parameter in different units of measurement, obtaining the value through a calibration curve. Here, we used an antigen solution based on the complete virus that was quantified by the TCID_50_ method, whereas most other detection systems use purified protein or genome copies. Under the reported conditions, our system has an estimated detection limit of 5.12 × 10^2^ PFU (see Figure 10). This estimated value was based on the performance of the test when we used diluted virus samples, showing that the LFA worked only when we added the undiluted sample (see Supplementary Material SI). Testing the LFA with 1:10 dilutions produces a negative result, suggesting that the tested antigen concentrations are near the limit of detection of the test.

Compared to other novel proposed systems, we have a comparable limit of detection to other antibody-based tests, such as observed in the Tamiflu-resistant Influenza virus detection system against neuraminidase with 5 × 10^2^ PFU [49] or the SARS-CoV-2 magnetic lateral flow system against the spike protein that showed a limit of detection (at the naked eye) of 6 × 10^2^ PFU [50]. As expected, the limit of detection of antibody-based systems is lower than that of nucleic acid amplification-based tests, which can reach a limit of detection of 2 × 10^2^ PFU [51].

The advantages of our test with a lateral flow format are its ease of use and minimal sample-handling steps, which are favorable for the environment. The functionality test showed that the results are not modified over time, preserving the signal in the detection window, which can be useful for capturing the results after the test. This LFA was designed to monitor flaviviruses in mosquito samples in order to identify areas of epidemic risk. The naked-eye readings will allow monitoring without equipment, making this method accessible for low-income regions.

We consider this prototype to be an advancement in the design of an accessible and easy-to-use LFA system for the detection of dengue virus and other flaviviruses but it is necessary to include other studies to determine its sensitivity and specificity. Functionality tests of the LFA with mosquito samples and the optimization and improvement of this LFA are required in order to use this LFA as a vector-monitoring tool. Among the improvements and optimizations of the design, different antibodies could be tested to determine if the sandwich format can improve the detection limit and the performance of the LFA.

## 5. Conclusions

In this study, a biosensor based on antibodies coupled to gold nanoparticles was developed and characterized for the detection of DENV, with the potential to detect other flaviviruses. The AuNP–4G2 biosensor showed physicochemical characteristics, which distinguishes it from naked AuNPs. Additionally, with this biosensor, a functional competitive LFA was designed and tested. The total time of the operation of this screening test, from the application of the sample on the LFA to obtaining the results, was a maximum of 10 min. The LFA functionality results showed that it is possible to detect DENV in vitro with this test.

Due to its performance and response times, this test proved to be a fast, easy, reliable, and low-cost alternative design for a point-of-care test for the monitoring of flaviviruses in areas endemic to the vector, both for early detection in mosquitoes and containment of possible outbreaks. This test prototype is expected to be used for the design of a commercial device for the rapid, sensitive, and specific mass screening of different samples for the accurate detection of DENV and other different flaviviruses, in order to increase primary prevention to avoid diseases caused by these viruses and decrease the consequences for vulnerable populations.

## Data Availability

Not applicable.

## Supplementary Materials

The following supporting information can be downloaded at: https://www.mdpi.com/article/10.3390/bios12070495/s1. Table S1: Equations and calculations to determine the TCID_50_ and PFU. Table S1 shows data for titration of virus stocks; Table S2: Determination of the hydrodynamic diameter (relative size), ζ-potential, and distribution (polydispersity index) of the AuNP conjugates.
