# Supplementary material for: Development of a Rapid Gold Nanoparticle-Based Lateral Flow Immunoassay for the Detection of Dengue Virus
Source: Biosensors (Basel). 2022 Jul 7;12(7):495. doi: 10.3390/bios12070495 (PMC9313084; doi:10.3390/bios12070495)
Supplement: Supplementary file 1 [file biosensors-12-00495-s001.zip › biosensors-1728240-supplementary.pdf]

Development of a Rapid Gold Nanoparticles-Based Lateral Flow Immunoassay for the Identification of Dengue Virus

Manuscript ID: biosensors-1728240

Supplementary Material

Supplementary Information 1. Equations and calculations to determine the $TCID_{50}$ and PFU.

Table S1 shows data for titration of virus stocks.

Table S1.

Log of virus dilution	Positive units	Total units	Ratio percent (A/B)*100
-1	6	6	100
-2	5	6	83.33
-3	2	6	33.33
-4	2	6	33.33
-5	1	6	16.66

With these results, the log viral titer was calculated by the Reed-Muench method [1], first calculating the proportionate distance;

$$Proportionate\ distance\ (PD) = \frac{\%\ mortality\ greater\ than\ 50\% -\ 50\%}{\%\ mortality\ greater\ than\ 50\% - \%\ mortality\ below\ 50\%}$$

$$PD = \frac{83.33 - 50}{83.33 - 33.33} = \frac{33.33}{50} = 0.66$$

To calculate the viral titer, it was determined as follows:

$$Negative\ logarithm\ of\ LD50\ endpoint\ titer = PD * \ negative\ Logarithm\ of\ factor\ dilution$$

$$= 0.66 * -1 = -0.66$$

$$\boldsymbol{Log\ LD50\ titer} = Negative\ logarithm\ of\ the\ lower\ dilution\ (next\ above\ 50\%\ mortality) - Negative\ logarithm\ of\ LD50\ endpoint\ titer$$

$$\boldsymbol{LD50\ titer} = (-2) + (-\mathbf{0.66}) = -\mathbf{2.66}$$

$$\boldsymbol{Log\ LD50\ titer = 10^{-2.66}}$$

From this value, the $TCID_{50}$/mL was determined with the following equation:

$$Infectious\ doses\ per\ unit\ volume = \frac{\frac{1}{\log LD50\ titer}}{viral\ inoculum\ volume}$$

$$= \frac{\frac{1}{10^{-2.66}}}{0.1\ mL}$$

$TCID_{50}$/mL= $\mathbf{4.58}\ \boldsymbol{x}\ \mathbf{10^3}$

The value $TCID_{50}$/mL was converted to plaque forming units per mL (PFU/mL) using the Poisson distribution [2-5]. This conversion is an estimate based on the rationale that the limiting dilution, which would infect 50% of the cell layers challenged, would be expected to produce a single plaque in a cell monolayer.

To estimate PFU/mL from $TCID_{50}$/mL, the Poisson distribution can be applied; P(o) is the proportion of negative tubes and 'm' is the mean number of infectious units per volume (PFU/mL),

$$P(o) = e(-m)$$

For any titer expressed as $TCID_{50}$, *P(o)*=0.5; *m*= -ln 0.5; therefore, the value of *e* is 0.693 ≈ 7 [2]. Then;

$$\boldsymbol{PFU/mL} = 4.58\ x\ 10^3 * (0.7) = \mathbf{3.2}\ \boldsymbol{x}\ \mathbf{10^3}$$

To estimate total PFU value, the PFU/mL was multiplied by the volume in mL used in the strip (since the undiluted stock was used);

$$\boldsymbol{Total\ PFU} = 3.2\ x 10^3 * 0.160 = 5.12\ x\ 10^2$$

Supplementary Table S2. Determination of the hydrodynamic diameter (relative size), ζ-potential and distribution (polydispersity index) of the AuNP conjugates.

**Sample**	**Hydrodynamic diameter**	**ζ -Potential**	**Polydispersity index**
AuNP CIT	67.69	-34.9	0.2

AuNP-BSA	103.5	-24.17	0.136
AuNP- 4G2 [1 µg]	107.87	-23.43	0.120
AuNP- 4G2 [3 µg]	117.20	-24.83	0.167
AuNP- 4G2 [6 µg]	106.63	-22.30	0.139

References

1. Horstmann, D. M. DIAGNOSTIC PROCEDURES FOR VIRAL AND RICKETTSIAL INFECTIONS (4th Ed.). Am. J. Public Heal. Nations Heal. 1970, 60 (5), 950–951.

2. The American Type Culture Collection (ATCC). Virology Guide. Available in https://www.atcc.org/-/media/resources/culture-guides/virology-guide.pdf?rev=a3816afe46cb499c90265fcf6456d2e3.

3. Logan, M., Manalil, J., Notte, C., Kearse, C., George, S., Zeiser, A., Farrell, P., & Aucoin, M. G. (2019). A flow cytometric granularity assay for the quantification of infectious virus. Vaccine, 37(47), 7090–7099. https://doi.org/10.1016/j.vaccine.2019.02.059

4. Manenti, A., Molesti, E., Maggetti, M., Torelli, A., Lapini, G., & Montomoli, E. (2021). The theory and practice of the viral dose in neutralization assay: Insights on SARS-CoV-2 "doublethink" effect. Journal of virological methods, 297, 114261. https://doi.org/10.1016/j.jviromet.2021.114261

5. Stanley, S., Hamel, D. J., Wolf, I. D., Riedel, S., Dutta, S., Contreras, E., Callahan, C. J., Cheng, A., Arnaout, R., Kirby, J. E., & Kanki, P. J. (2022). Limit of Detection for Rapid Antigen Testing of the SARS-CoV-2 Omicron and Delta Variants of Concern Using Live-Virus Culture. Journal of clinical microbiology, 60(5), e0014022. https://doi.org/10.1128/jcm.00140-22
